# The relationship between sleep disorders and frailty in stroke patients: the mediating role of self-efficacy

**DOI:** 10.3389/fpsyt.2025.1565412

**Published:** 2025-06-26

**Authors:** Shuyuan Niu, Manjiang Liu, Yingjie Lin, Peiqi Gu, Li Zhao

**Affiliations:** School of Geriatrics and Elderly Care Industry, Shenyang Medical College, Liaoning, Shenyang, China

**Keywords:** stroke patients, sleep disorders, self-efficacy, frailty, structural equation model

## Abstract

**Background:**

Stroke patients may have symptoms such as sleep disorders, and the incidence of frailty is much higher than that of non-stroke patients. Self-efficacy can reduce the occurrence of frailty and sleep disorders and help to maintain the health of stroke patients.

**Objective:**

To investigate the relationship between sleep disorder, self-efficacy and frailty in stroke patients, and to analyse the mediating role of self-efficacy.

**Methods:**

This study was a cross-sectional study, and 6 “stroke map” sites were selected in Shenyang from June to September 2024. A total of 924 stroke patients were included, and a structural equation model (SEM) was constructed to analyse the mediating effect of self-efficacy on sleep disorders, self-efficacy and frailty in stroke patients.

**Results:**

The prevalence of debilitation in stroke patients was 46.2%. Sleep disorders were positively correlated with frailty (*P*<0.01) and negatively correlated with self-efficacy. Self-efficacy is negatively correlated with frailty, which is the intermediary between sleep disorders and frailty.

**Conclusion:**

The prevalence of frailty in stroke patients is high. Sleep disorders are highly correlated with frailty, and self-efficacy plays a mediating role between sleep disorders and frailty, which can reduce the impact of sleep disorders on frailty development.

## 1 Introduction

Stroke is one of the major causes of death and disability in the world. The incidence risk of stroke in China is as high as 39.9%, ranking first in the world (1). Stroke patients may have a variety of symptoms, including cognitive Frailty, sleep disorders, movement limitations and weakness (2–4). Studies have shown that stroke patients are more likely to suffer from frailty than non-stroke patients (5). Frailty is an intermediate state between health and disease, reflecting the Frailty of human physiological reserve capacity and the increase of susceptibility to stressors. The core features of frailty are the significant Frailty of physiological reserve capacity, the increase of body vulnerability and the weakening of resistance to external stressors (6, 7). The existence of frailty makes even small external stimuli likely to trigger serious clinical events, especially in the management of stroke patients. Frailty further aggravates the complexity of disease management and brings more severe challenges to medical care (8). Thus, frailty is an important research area in the field of public health and clinical prognosis.

Studies have shown that the incidence of debilitation in stroke patients is as high as 66.8% (9). The incidence of frailty in hospitalised stroke patients aged 65 years and older is 6 times that of non-stroke patients (10). Because of the occurrence of patients with frailty, many investigations have been carried out at home and abroad, with different results. A series of studies conducted by domestic scholars have shown that the prevalence rate of elderly stroke patients in China is 86.9% (11, 12), while relevant studies by foreign scholars have shown that the incidence of debilitation in stroke patients is about 60% (13, 14). The large differences in the incidence of frailty at home and abroad may be related to the differences in disease types, assessment tools, sample size, etc., but the overall incidence of frailty is relatively high (15). This may be due to the direct or indirect effects of A variety of risk predictors, including hypersensitive C-reactive protein, serum amyloid A, soluble CD40 ligand, and systemic immuno-inflammatory index in stroke patients (16).

Previous studies have shown that sleep disorders are positively correlated with the prevalence of frailties in stroke patients (17). Sleep disorders refer to abnormal sleep volume and abnormal alterations of sleep and wakefulness, which often occur after stroke and involve various forms and interfere with the functional life of stroke patients (18). Sleep disorders may lead to decreased glucose tolerance and overexcitation of the sympathetic nerve, thus adversely affecting the regulation mechanism of blood sugar and blood pressure, further hindering the recovery of neurological function and affecting the prognosis of stroke patients (19). Relevant studies have shown that compared with stroke patients without sleep disorders, patients with sleep disorders show more serious symptoms in terms of neurological impairment, which not only significantly affects the functional rehabilitation of patients but also increases the risk of stroke recurrence (20). Quality sleep is crucial to People’s Daily life, cognitive function and endocrine function, so sleep disorders will have multiple impacts on individuals’ physical and emotional health (21). Studies have shown that sleep disorders are common in stroke patients, with an incidence of about 78% (22, 23). Sleep disorders are not only related to the rehabilitation outcome of stroke patients, but also the debilitation of stroke patients, so sleep disorders should be considered in the treatment of stroke patients to reduce the debilitation.

Social cognition theory is a psychological theory proposed by the psychologist Albert Bandura in the 1960s. This theory mainly studies how individuals learn social behaviour through observation, imitation and interaction, and emphasises the importance of cognitive processes in behaviour learning. Its core concepts mainly include ternary interactive determinism, observational learning, self-efficacy and cognitive process (24). Among them, self-efficacy, as the core concept of the theory, is rooted in the core idea of behavioural psychology. It not only attaches importance to the in-depth study of human behaviour but also emphasises the principle of objectivity of research and the importance of reinforcement in the learning process. At the same time, self-efficacy also explores the mysteries of internal psychological processes, especially highlighting the mediating role of self-factors in behaviour. Several studies have shown that patients with high self-efficacy can adhere to exercise, reduce weakness, and significantly improve the quality of life of patients (25, 26). Self-efficacy plays a key role in the relationship between sleep disorders and frailty in stroke patients. It can effectively regulate the frailty of patients. Enhancing the confidence and positive expectations of stroke patients in their abilities can stimulate the motivation of stroke patients to adopt positive coping behaviours, thereby reducing their frailty symptoms and promoting the recovery of physical and mental health.

There are many studies on the related factors of frailty in stroke patients, but there are relatively few studies on the role of positive psychology, such as self-efficacy, in the relationship between sleep disorders and frailty. Therefore, the purpose of this study was to elucidate the relationship between sleep disorders and frailty in stroke patients and to explore whether self-efficacy plays a mediating role between sleep disorders and stroke patients.

## 2 Materials and methods

### 2.1 Study design and sample

Stroke patients in six hospitals designated by the “Stroke Map” in Shenyang were selected as research objects in Shenyang, including Shenyang First People’s Hospital, the Second Affiliated Hospital of Shenyang Medical College, Shenyang Third People’s Hospital, Shenyang Fourth People’s Hospital, Shenyang Fifth People’s Hospital and Shenyang Seventh People’s Hospital (27). Inclusion criteria: Patients diagnosed with ischemic or hemorrhagic stroke with a clinician and neuroimaging support; Stable patients in the recovery phase whose most recent stroke occurred within four weeks; Able to understand spoken or written language and able to respond using words or gestures; Informed consent. Exclusion criteria; Serious medical condition (such as massive cerebral infarction or bleeding, severe myocardial infarction or heart surgery, etc.); Pre-existing neurological disease (Parkinson’s disease, multiple sclerosis or severe dementia, etc.); Serious uncontrolled mental illness or acute infectious disease; Persistent history of alcohol or drug abuse in the past six months.

In this study, questionnaires were distributed using the questionnaire star platform (28). According to the Kendall multi-factor study’s sample size estimation method, the sample size should be 5–10 times the number of studied variables (29). There are 44 variables in this study. Considering the invalid questionnaire, the sample size should be expanded by 10% based on the original sample size, so the sample size investigated should be at least 484 people. To improve the statistical efficiency, this study expands the sample size to 1000 people. A total of 924 qualified questionnaires were recovered. The response rate is 92.4%.

### 2.2 Measures

#### 2.2.1 Measurement of socio-demographics

Data on socio-demographics were collected from all participants, including gender, ethnicity, height, weight, occupation, age, marital status, education level, residence area, family income, chronic disease, stroke type, thrombolytic therapy, smoking, drinking, etc.

#### 2.2.2 Measurement of frailty

The Tilburg frailty indicator (TFI) was used to assess the severity of frailty. The scale was developed by Gobbens et al. (2010) based on integrated debilitation models, and it was Sinicised by Xi Xing et al. (30, 31). The TFI scale consists of 3 dimensions and 15 items. The score range is 0~15 points. ≥5 points indicate frailty. The higher the score, the more serious the frailty degree. Cronbach’s α coefficient of the frailty scale in this study was 0.896.

#### 2.2.3 Measurement of self-efficacy

The general self-efficacy scale (GSES) was compiled by Schwarzer et al., using Likert’s 4-point scoring method, ranging from 1 point (very inconsistent) to 4 points (very consistent), with a total of 10 questions and 1 dimension. The higher the cumulative score, the higher the sense of self-efficacy (32). In this study, the Cronbach’s α coefficient of the general self-efficacy scale was 0.972.

#### 2.2.4 Measurement of sleep disorders

The present study adopted the Chinese version of the Pittsburgh Sleep Quality Index (PSQI) to evaluate the sleep disorders of patients with Stroke in the last month. This scale was compiled in 1989 by Buysse, University of Pittsburgh in the United States, to assess the sleep status of the measured person in the past month, and it was translated into Chinese by domestic scholar Liu Xianchen et al. (33). The scale consists of 18 items and 7 dimensions. The PSQI is the total score of 7 dimensions, ranging from 0 to 21 points. A higher total score indicates poorer sleep quality and a score of ≥7 indicates a sleep disorder. In this study, Cronbach’s α coefficient of the sleep disorder scale was 0.799 (34).

### 2.3 Statistical analysis

**Figure 1:**
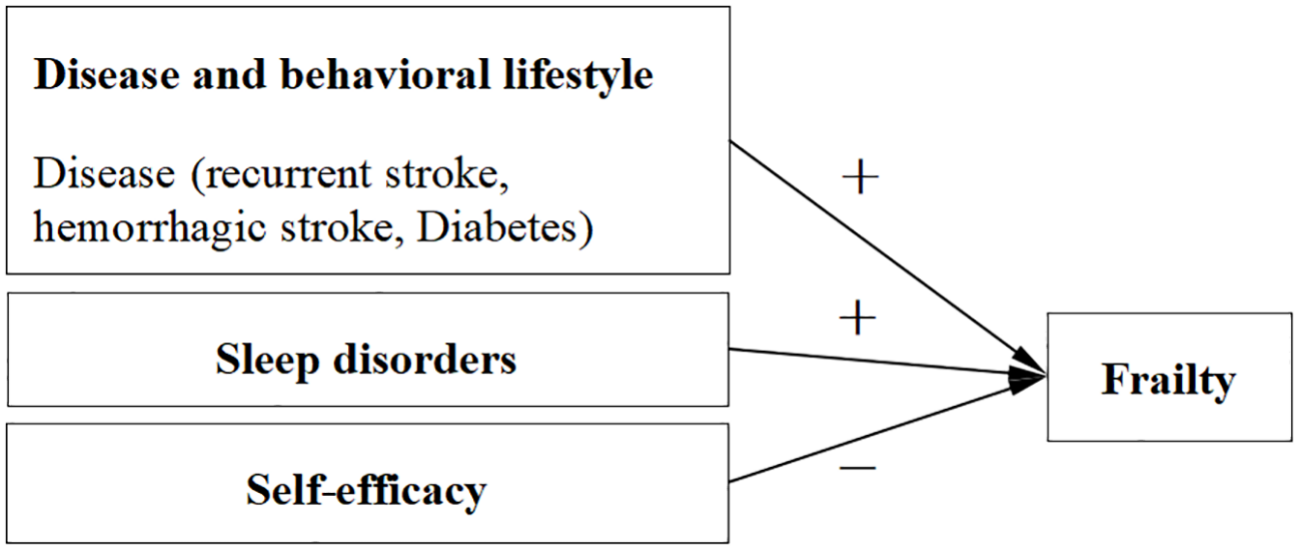
Factors influencing frailty in stroke patients.

Analysis of structural equation structure, AMOS 24.0 and SPSS 24.0 software were used to analyse the data. The independent sample T-test and one-way analysis of variance (ANOVA) were used to compare the differences in frailty among different categorical variables. The T-test of two independent samples was used to compare the differences of stroke patients in sex, age, nationality, marital status, education level, marital status, working condition, per capita monthly household income, Home location, chronic disease status, recurrent stroke, hypertension, smoking, and drinking (**Figure 1**). Single-factor ANOVA was used to investigate the differences among multiple categorical variables, such as stroke type. Spearman correlation analysis was used to examine the correlation between sleep disorders, self-efficacy, and frailty in stroke patients. Hierarchical regression (HMR) was used to explore the influencing factors of frailty. The basic condition index with statistical significance in univariate analysis was taken as the first layer, the second layer was put into sleep disorders, and the third layer was put into self-efficacy to explore the role of each factor in frailty. Using AMOS 24.0, the Structural Equation Model (SEM) (35) was used to construct the relationship model between sleep disorders, self-efficacy and frailties in stroke patients.

We first incorporated the sleep disorders and debilitation scale into SPSS for exploratory factor analysis, and the results showed that the dimensions of the two scales in this study were consistent with the original dimensions. Then we used AMOS for confirmatory factor analysis of the two questionnaires and obtained a good fitting index of the questionnaire. We incorporated the dimensions of sleep disorder and weakness into the structural equation model for analysis. The reliability and validity of the three questionnaires are satisfactory, and the maximum likelihood method was used to estimate the parameters. The model conforms to SEM criteria (χ^2^/df=3.820, RMSEA=0.055, CFI=0.938, IFI=0.939, TLI=0.933). For the mediation effect test, the Bootstrap method was used to sample 5000 times repeatedly, and the deviation correction and 95% CI of the model path a*b were checked. P<0.05 indicated that the difference was statistically significant.

## 3 Results

### 3.1 Demographic characteristics

The confounding variables in this study are divided into three categories. Demographic factors include sex, age, nation, educational level, marital status, working condition, per capita monthly household income and home location. Behavioural factors include drinking and smoking. Clinical factors include hypertension, hyperlipemia, diabetes, coronary heart disease, recurrent stroke, thrombolysis and stroke types. Three core variables were included as y-values in the regression analysis, and confounding variables were included as x-values. The specific results are shown in **Table 1**. Among these patients, 542 participants were male (58.7%), and the patients aged 60 and above accounted for 82.1%. Most of them were Han Chinese, accounting for 88.9%, and the majority of those with a high school education or below accounted for 743. Married (88.1%) and retired (80.5%) make up the vast majority. There were 517 patients with a per capita monthly household income of 4,000 or less, and most of the patients were registered in cities (74.1%). Among the stroke patients in this study, 509 were drinkers(55.1%), and 578 were smokers(62.6%). Chronic conditions include hypertension(60.9%), hyperlipidemia(20.6%), diabetes(22.8%), coronary heart disease(14.8%).There were 484 patients with a first stroke(52.4%), and 270 patients with thrombolysis. As for the stroke types, there are 148 patients with TIA, 680 patients with ischemic stroke and 96 patients with hemorrhagic stroke.

**Table 1:** Demographic characteristics and distributions of different data groups.

Group	Number/%	Sleep disorders	Self-efficacy	Frailty
Sex				
Male	542 (58.7%)	7.28 ± 4.21	26.51 ± 9.19	5.23 ± 4.39
Female	382 (41.3%)	7.32 ± 4.13	26.33 ± 9.91	5.78 ± 4.77
Age				
<60	165 (17.9%)	7.21 ± 3.95	28.06 ± 9.31^*^	5.07 ± 4.57
≥60	759 (82.1%)	7.32 ± 4.22	26.08 ± 9.5	5.54 ± 4.55
Nation				
Han Chinese	821 (88.9%)	7.31 ± 4.19	26.38 ± 9.42	5.46 ± 4.55
Other nationalities	103 (11.1%)	7.22 ± 4.05	26.85 ± 10.04	5.42 ± 4.65
Educational level				
High school and below	743 (80.4%)	7.39 ± 4.26	26.26 ± 9.4	5.56 ± 4.62
Above high school	181 (19.6%)	6.91 ± 3.81	27.13 ± 9.86	5.04 ± 4.27
Marital status				
Be married	814 (88.1%)	7.22 ± 4.1	26.55 ± 9.56	5.44 ± 4.57
Others	110 (11.9%)	7.85 ± 4.66	25.56 ± 8.96	5.6 ± 4.48
Working condition				
Retirement	744 (80.5%)	7.25 ± 4.18	26.14 ± 9.56	5.55 ± 4.6
Others	180 (19.5%)	7.49 ± 4.18	27.64 ± 9.13	5.06 ± 4.35
Per capita monthly household income				
≤4000	517 (56%)	7.24 ± 4.23	26.1 ± 9.13	5.29 ± 4.47
>4000	407 (44%)	7.38 ± 4.11	26.85 ± 9.93	5.67 ± 4.66
Home location				
Rural	239 (25.9%)	7.6 ± 3.98	25.9 ± 9.78	5.93 ± 4.78
City	685 (74.1%)	7.19 ± 4.24	26.62 ± 9.39	5.29 ± 4.47
Drinking				
Yes	415 (44.9%)	7.93 ± 4.36^**^	23.32 ± 9.09	7.01 ± 4.74^**^
No	509 (55.1%)	6.79 ± 3.95	28.97 ± 9.05^**^	4.19 ± 3.98
Smoking				
Yes	346 (37.4%)	8.61 ± 4.73^**^	22.71 ± 9.63	6.92 ± 4.89^**^
No	578 (62.6%)	6.52 ± 3.59	28.66 ± 8.67^**^	4.58 ± 4.1
Hypertension				
Yes	563 (60.9%)	7.21 ± 4.2	26.2 ± 9.4	5.44 ± 4.47
No	361 (39.1%)	7.44 ± 4.14	26.79 ± 9.63	5.48 ± 4.69
Hyperlipemia				
Yes	190 (20.6%)	7.15 ± 4.08	26.08 ± 10.21	5.15 ± 4.35
No	734 (79.4%)	7.34 ± 4.2	26.52 ± 9.3	5.54 ± 4.6
Diabetes				
Yes	211 (22.8%)	7.21 ± 4.29	25.12 ± 9.33	5.14 ± 4.08
No	713 (77.2%)	7.33 ± 4.14	26.82 ± 9.51^*^	5.55 ± 4.68
Coronary heart disease				
Yes	137 (14.8%)	7.61 ± 4.26	25.15 ± 9.47	5.32 ± 4.42
No	787 (85.2%)	7.25 ± 4.16	26.66 ± 9.48	5.48 ± 4.58
Recurrent stroke				
Yes	440 (47.6%)	8 ± 4.41^**^	24.02 ± 9.75	6.81 ± 4.92^**^
No	484 (52.4%)	6.66 ± 3.84	28.63 ± 8.69^**^	4.23 ± 3.81
Thrombolysis				
Yes	270 (29.2%)	8 ± 4.54^**^	22.72 ± 8.17	6.41 ± 4.14^**^
No	654 (70.8%)	7.01 ± 3.99	27.96 ± 9.58^**^	5.06 ± 4.66
Stroke types				
TIA	148 (16%)	7.58 ± 4.12	29.06 ± 9.35^**^	5.17 ± 3.97
Ischemic stroke	680 (73.6%)	7.22 ± 4.21	26.48 ± 9.42	5.56 ± 4.67
Hemorrhagic stroke	96 (10.4%)	7.44 ± 4.04	22.01 ± 8.66	5.2 ± 4.56

^*^ *P<*0.05, ^**^ *P<*0.01.

There were statistical differences in sleep disorder scores between drinking, smoking, recurrent stroke and thrombolysis (*P*<0.05). Age, smoking, drinking, diabetes, recurrent stroke, thrombolysis and stroke type had significant differences in self-efficacy scores (*P*<0.05). Whether drinking, smoking, hypertension, recurrent stroke and thrombolysis had significant differences in frailty scores (*P*<0.05).

### 3.2 Correlations among sleep disorders, self-efficacy and frailty

Spearman correlation analysis was used to analyze the scales of self-efficacy, sleep disorders and frailty. There was a significant negative correlation between self-efficacy and sleep disorders (*P*<0.01), a significant negative correlation between self-efficacy and frailty, and a significant positive correlation between sleep disorders and frailty. As shown in **Table 2**.

**Table 2:** Correlation analysis of each scale.

	1	2	3
Sleep disorders	1		
Self-efficacy	-0.331^**^	1	
Frailty	0.413^**^	-0.560^**^	1

^**^ *P<*0.01.

### 3.3 Hierarchical multiple regression analysis of sleep disorders, self-efficacy and frailty

The HMR models of PSD are depicted in **Table 3**. Age, alcohol consumption, smoking, hypertension, diabetes, recurrent stroke, thrombolytic therapy, and stroke type. With statistical differences in the univariate analysis included as control variables in the first layer regression analysis, the Adjusted *ΔR^2^* was 0.194. The final HMR model explained a total of 19.4% of the variance in frailty.

**Table 3:** Hierarchical multiple regression analysis.

Variable		Layer 1	Layer 2	Layer 3
Basic situation	Age (<60vs≥60)	0.012	0.013	-0.016
	Drinking (No vs Yes)	0.238^**^	0.215^**^	0.122^**^
	Smoking (No vs Yes)	0.157^**^	0.091^**^	0.013
	Diabetes (No vs Yes)	0.036	0.034	0.065^**^
	Recurrent (No vs Yes)	0.228^**^	0.189^**^	0.117^**^
	Thrombolysis (No vs Yes)	0.111^**^	0.081^**^	-0.016
	Type (TIA vs Ischemic vs Hemorrhagic)	-0.029	-0.028	0.103^**^
Sleep disorders			0.325^**^	0.211^**^
Self-efficacy				-0.481^**^
R^2^		0.194	0.290	0.445
*Δ*R^2^		0.194	0.096	0.160
Adjusted R^2^		0.187	0.284	0.445
Adjusted *Δ*R^2^		0.187	0.097	0.161

**P<0.01.

In the second step, sleep disorders were added, and the results showed that sleep disorders were positively correlated with frailty (*β*=0.325, *P*<0.01). Adjusted *ΔR^2^* was 0.097, that is, 9.7% of frailty could be explained by sleep disorders. After adding self-efficacy in the third step, self-efficacy was negatively correlated with frailty score (*β*=-0.481, *P*<0.01). Adjusted *ΔR^2^* was 0.161, that is, 16.1% of frailty could be explained by self-efficacy. Hierarchical regression analysis can be used to assess the unique contribution of sleep disorders and self-efficacy to frailty while gradually controlling for the covariates.

### 3.4 Structural equation modelling results

**Figure 2:**
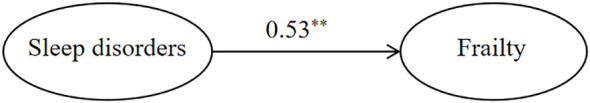
Model of direct effects of sleep disorders and frailty.

The direct effect model between sleep disorders and frailty is shown in **Figure 2**. Structural equation model results show that sleep disorders have a significant direct impact on frailty in stroke patients (*c*=0.53, *P*<0.01). The structural equation model showed a positive correlation between sleep disorder and then, with a good model fitting index (*χ^2^/df*=4.905, RMSEA=0.065, CFI=0.915, IFI=0.915, TLI=0.904).

**Figure 3:**
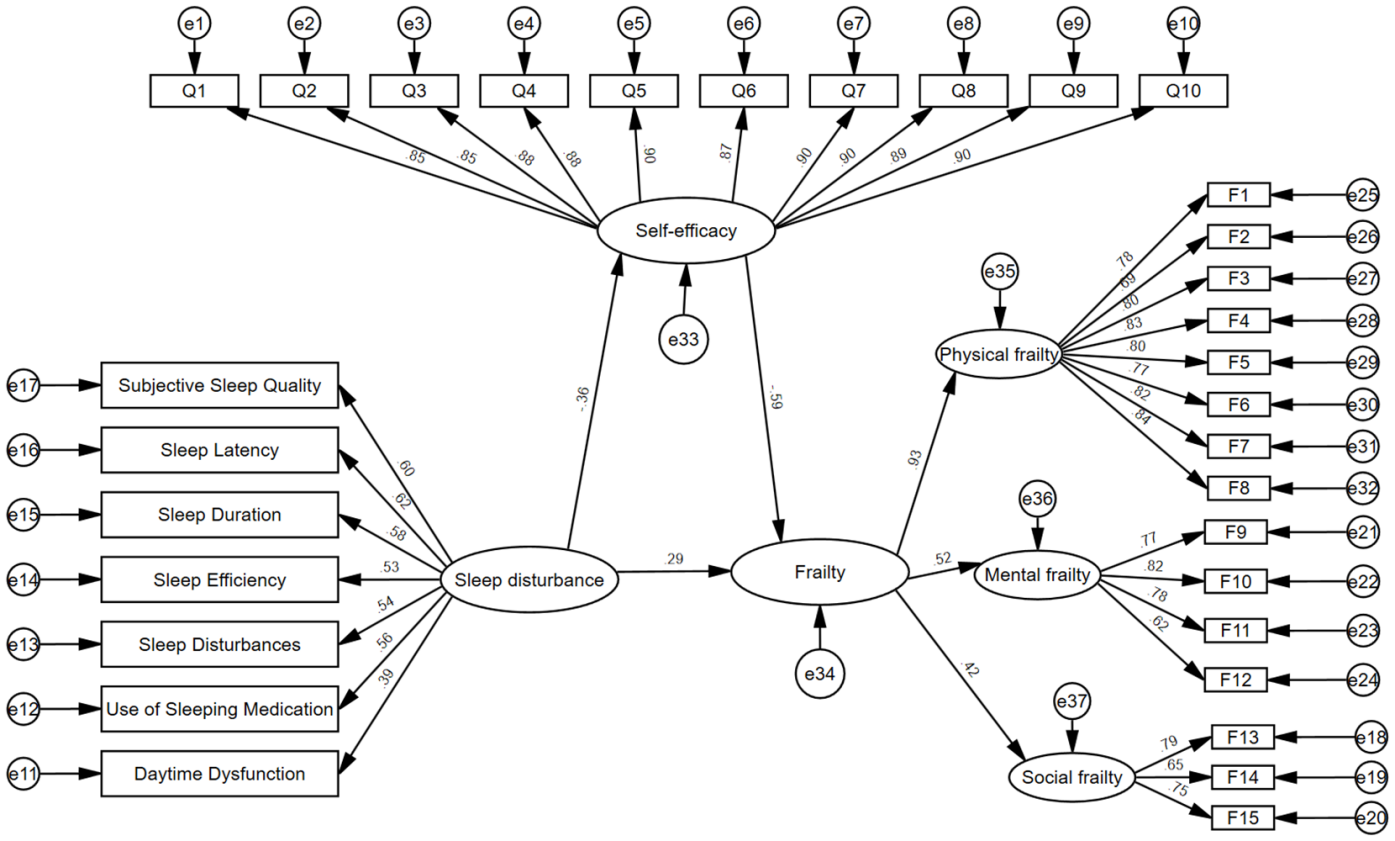
Structural equation model of the relationship between sleep disorders, self-efficacy and frailty.

The mediating role of self-efficacy between sleep disorders and frailty is shown in **Figure 3**. The dimension of each variable has also been shown in the figure. There was a significant negative correlation between self-efficacy and frailty (*β*=-0.59, *P*<0.01). When self-efficacy was used as an intermediary, the path coefficient between sleep disorders and frailty was significantly reduced (*C ‘* = 0.29, *P*<0.01). Bootstrap (*N*=5000) method was used to test the parameters and mediating effects. The results showed that (a*b=0.1, *95%CI:* 0.07-0.11), the confidence interval did not contain 0. The model fit index was up to standard (*χ^2^/df*=3.820, RMSEA=0.055, CFI=0.938, IFI=0.939, TLI=0.933). It can be concluded that sleep disorders affect frailties both directly and indirectly through the mediating pathway of self-efficacy (^**^represents significant path coefficient).

### 3.5 SEM analysis results of the impact of different sleep problems on the weakness of stroke patients

**Figure 4:**
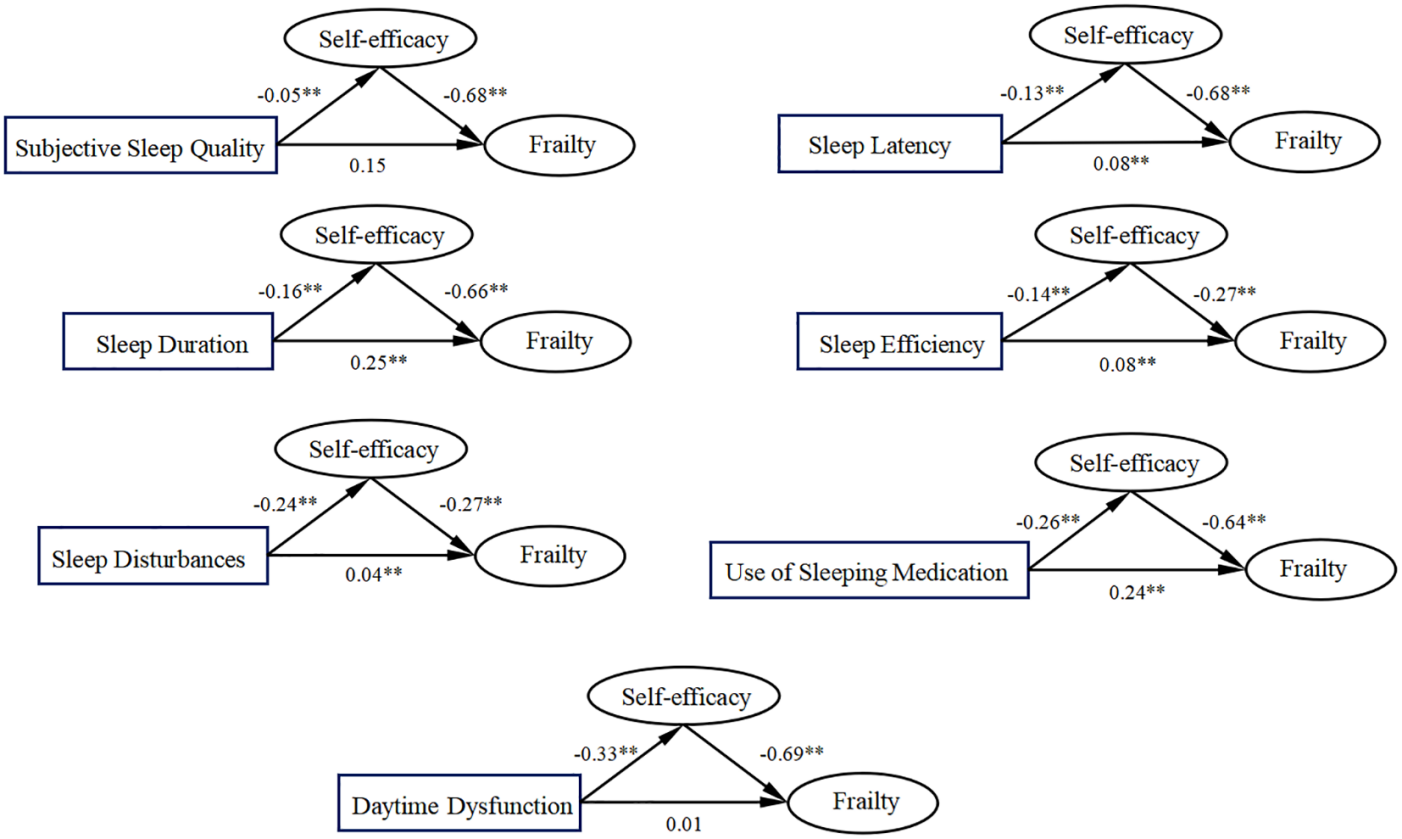
Results of multidimensional analysis of sleep disorders.

In this study, the seven dimensions of sleep disorders were taken apart to construct the structural equation model. The results are shown in **Figure 4**, and the specific indicators are shown in **Table 4**. The seven components of the PSQI assessment also showed different correlations with self-efficacy and weakness, and the correlations were all positive. These results further confirmed the correlation among sleep disorders, self-efficacy and weakness.

**Table 4:** Structural equation model analysis of sleep disorders.

Dimensions of sleep disorders	*β*	*SE*	*P*
Subjective Sleep Quality	0.048	0.014	0.059
Sleep Latency	0.079	0.001	0.005
Sleep Duration	0.251	0.011	0.001
Sleep Efficiency	0.076	0.009	0.001
Sleep disorders	0.041	0.010	0.001
Use of Sleeping Medication	0.242	0.010	0.001
Daytime Dysfunction	0.012	0.011	0.693

## 4 Discussion

### 4.1 Prevalence of frailty and sleep disorders in stroke patients

This study explored the relationship between sleep disorders and frailty, and the mediating role of self-efficacy between the two. The study results showed that 46.2% of stroke patients had frailty, which was consistent with the study results of Hanlon et al., 23.8%-54.6% (36). However, compared with the results of Taylor-Rowan et al. ‘s study of 28%, the prevalence rate in this study was higher (37). This difference may be because Taylor-Rowan et al. ‘s study mainly focused on patients with acute stroke, and included some patients who were at risk of the disease, whose condition was not stable or who were in the recovery stage. This results in a lower prevalence of frailty, so the findings of this study can more comprehensively reflect the prevalence of frailty in stroke patients. In addition, in this study, the incidence of sleep disorders in stroke patients was 47.9%, which was significantly higher than that of Karaca B et al., who used PSQI as a research tool to study the prevalence of stroke patients (39.1%) (38). This difference can be attributed to the larger sample size used in this study, which reduced sampling error and improved the representativeness and accuracy of the findings, thereby revealing more fully the prevalence and severity of sleep disorders in the stroke patient population.

### 4.2 Heterogeneity in self-efficacy among stroke patients: a comparative analysis

This study found that age, smoking, drinking, diabetes, stroke recurrence, thrombolysis, and stroke type were significantly different in self-efficacy scores. Specifically, the self-efficacy scores of patients with advanced age, smoking, drinking, diabetes, stroke recurrence, thrombolytic therapy and hemorrhagic stroke were significantly lower. It may be because the decrease in self-efficacy in elderly patients may be related to the decrease in physiological function, The increasing burden of chronic diseases and diminished rehabilitation self-efficacy pose significant clinical challenges. Empirical evidence indicates that elderly stroke patients frequently present with multiple comorbidities, which may contribute to impaired recovery motivation and compromised functional outcomes, which may weaken their confidence in dealing with the disease (39). In addition, age-related cognitive decline may affect patients ‘ ability to understand and implement rehabilitation strategies. Studies have shown that the neurotoxicity of nicotine and alcohol may damage prefrontal function and weaken decision-making ability and goal persistence.

### 4.3 Drinking, diabetes, recurrent stroke and hemorrhagic stroke are the factors affecting the debilitation of stroke patients

By using the independent sample T-test to analyze the difference in control variables, it was found that smoking, drinking, recurrence, thrombolysis and stroke type had significant differences in the scores of the three scales, indicating that behaviour, lifestyle and disease status had a great impact on the physical and mental status of patients. This study did not find significant differences in frailty scores among stroke patients with different social backgrounds. Although previous studies have suggested that female stroke patients may have different psychological adaptations due to differences in hormone levels or social roles (40), gender did not show a significant effect in this study. It may be due to the low proportion of women in the sample or family support in the cultural background that buffers gender differences. Marital status and place of residence did not significantly affect the results, which may be related to the high proportion of married and urban residents in the sample. The economic level and occupational status did not show the expected impact, suggesting that economic pressure may be partially alleviated in the context of better medical insurance coverage (41).

Regression analysis showed that patients with alcohol consumption, diabetes mellitus, recurrent stroke, and hemorrhagic stroke had more severe frailty. This may be because drinking alcohol can increase the incidence of frailty in stroke patients through the combined effects of increasing blood pressure, promoting blood clotting, reducing cerebral blood flow, and interfering with drug efficacy and neurotoxicity (42). Combined diabetes can cause skeletal muscle mitochondrial dysfunction and microvascular disease in patients, resulting in debilitation of patients (43). For patients with recurrent stroke, the underlying pathological factors of stroke occurrence have not been removed, which increases the complexity and difficulty of patients’ self-management and also poses a potential risk. Studies have shown that up to 50% of stroke patients lack sufficient understanding of recurrence risk and cannot accurately perceive the actual risk of stroke recurrence. This makes recurrent stroke patients more vulnerable to disease management and thus more prone to frailty (44). Due to brain tissue injury, nerve cell death brain oedema and other pathophysiological mechanisms, hemorrhagic stroke patients are more likely to experience motor, speech, cognitive and sensory dysfunction, and thus become debilitating.

### 4.4 Sleep disorders are positively affecting the frailty of stroke patients

Spearman correlation analysis results showed a significant positive correlation between sleep disorders and frailty, that is, the more severe sleep disorders, the higher the degree of frailty of patients, which is consistent with previous relevant studies (45, 46). The results of regression and structural equation modelling show that sleep disorders have a direct impact on frailty occurrence in Chinese stroke patients, and can directly lead to an increased risk of frailty occurrence. The possible reason is that the daily living ability of stroke patients is usually seriously affected, such as mobility difficulties, self-care ability Frailty, etc. These dysfunctions will limit the range of activities of patients, make patients often suffer from anxiety, depression and other emotional problems, increase their psychological burden, and affect the quality of sleep of patients. However, poor sleep quality will cause the Frailty of patients’ nervous system function, further aggravate patients’ mental and psychological pressure, lead to the obstruction of patients’ physical repair, and accelerate the Frailty of physical function and the emergence of frailty (47). Therefore, good sleep is essential for maintaining individual cognitive function, promoting daily activities and maintaining mental health. For the improvement of sleep in stroke patients, the primary task is to optimise their overall health. It is recommended that patients follow a healthy and regular lifestyle. At the same time, medical staff should comprehensively consider the age, body and living conditions of patients, and customise a personalised sleep disorder intervention program to effectively improve the sleep quality of patients.

### 4.5 Self-efficacy is negatively affecting the frailty of stroke patients

Spearman correlation analysis showed a significant negative correlation between self-efficacy and frailty in stroke patients (*P*<0.01), that is, the higher the score of self-efficacy, the lower the degree of frailty. The results of regression and structural equation modelling show that self-efficacy has a direct impact on frailty occurrence in Chinese stroke patients and can directly reduce the risk of frailty occurrence. At present, the treatment of stroke patients in comprehensive hospitals in China mostly focuses on physical rehabilitation and pays less attention to patients’ physical state and mental health, which leads to the reduction of patient’s quality of life and poor prognosis. The perception of self-efficacy is rooted in the theoretical framework of social cognition, which reflects the self-assessment of an individual’s ability and belief to carry out specific behaviours needed to achieve goals, and individuals with low self-efficacy have significant restorative effects on physical behaviours and functions (48). Studies have shown that elderly people with high self-efficacy can accurately and objectively perceive their self-worth, enhance their autonomy in physical activities, enable them to deal with various challenges in life with a more confident attitude, and continuously improve their social life participation through positive social interaction, thus having a positive impact on health. It can help delay the Frailty of physical function and frailty of the elderly (49). Therefore, to better rehabilitate stroke patients, the self-efficacy of stroke patients should be strengthened.

### 4.6 Self-efficacy plays a mediating role between sleep disorders and frailty

It is worth noting that in this study, sleep disorders not only have a direct effect on frailty in stroke patients but also have an indirect effect on frailty through the mediating pathway of self-efficacy. The association between sleep disorders and frailty in stroke patients confirms that the greater the sleep disorders, the higher the risk of frailty. Frailties may be more likely to occur in stroke patients with sleep disorders, but this association may be reversed by increased self-efficacy, the study showed. Self-efficacy can effectively regulate sleep quality, reduce the adverse effects of sleep quality on frailty, and delay frailty. Self-efficacy can affect frailty directly or indirectly by reducing loneliness. Studies have shown that self-efficacy plays a mediating role in the relationship between social support and daily activity ability (50). Self-efficacy can prevent frailty, promote patients’ ability to combat the effects of sleep disorders and enhance their ability to promote patients’ mental health. Therefore, clinical attention should also be paid to the self-efficacy of stroke patients to counter the stress situation, promote the process of rehabilitation, and improve the mental health status and quality of life of stroke patients.

### 4.7 Influence of different classification of sleep disorders on the frailty of stroke patients

Stroke patients often have difficulty falling asleep, which may be related to post-stroke pain, anxiety or depression, thus affecting the rehabilitation process of patients. Low sleep efficiency indicates that patients cannot get enough rest at night, which will further affect their ability of daily activities and the rehabilitation effect (51). Patients with poor sleep quality usually show daytime sleepiness, inattention and other problems, which will harm the rehabilitation of patients. In this study, the seven dimensions of sleep disorders were separated to construct structural equation models. It was concluded that the use of hypnotics and sleep duration had a greater impact on asthenia, with b values of 0.24 and 0.25, respectively. The shortening of sleep time may further aggravate the fatigue and cognitive impairment of patients, resulting in a poor rehabilitation effect. The use of hypnotic drugs will also exacerbate the weakness of stroke patients. The reason may be that the use of hypnotic drugs may lead to drug dependence in patients, and even trigger rebound insomnia and anxiety symptoms after withdrawal, aggravating the weakness of patients (52). There was no significant difference in the impact of subjective sleep quality and daytime dysfunction on frailty.

## 5 Conclusion

In this study, the incidence of frailty in stroke patients was associated with recurrent stroke, hemorrhagic stroke, diabetes, alcohol consumption, sleep disorders and self-efficacy. Sleep disorders were positively correlated with frailty, while self-efficacy was negatively correlated with frailty. Self-efficacy plays a mediating role in the relationship between sleep disorders and frailty and can reduce the negative impact of sleep disorders on frailty occurrence. Therefore, in clinical practice, attention should be paid to patient’s sleep quality and self-efficacy, and intervention guidance should be given according to the actual situation of patients, to promote the recovery of patients’ physical functions and to maintain their mental health, to reduce the occurrence of frailty.

## 6 Limitations

There are some limitations to the current study. This study is a cross-sectional study, and there is selection bias, resulting in the characteristics of the study sample not representing the whole target population. Second, only one city was selected for this study, which may limit the extensibility of the study. Finally, This study did not perform a more detailed classification of stroke types or account for the impact of stroke location and specific type on patient outcomes. Subsequent research will shift its focus to this area to address these limitations.

## Data Availability

The raw data supporting the conclusions of this article will be made available by the authors, without undue reservation.
