# Supplementary material for: The relationship between sleep disorders and frailty in stroke patients: the mediating role of self-efficacy
Source: Front Psychiatry. 2025 Jun 26;16:1565412. doi: 10.3389/fpsyt.2025.1565412 (PMC12240965; doi:10.3389/fpsyt.2025.1565412)
Supplement: Supplementary file 1 [file SupplementaryFile1.pdf]

The formative scale used for data collection

Tilburg frailty indicator（TFI）

Items of the TFI	Categorical variables
1.Do you feel physically healthy?	Yes=0<br>No=1
2.Have you lost a lot of weight recently without wishing to do so?	Yes=1<br>No=0
3.Do you experience problems in your daily life due to difficulty in walking?	Yes=1<br>No=0
4.Do you experience problems in your daily life due to difficulty maintaining your balance?	Yes=1<br>No=0
5.Do you experience problems in your daily life due to poor hearing?	Yes=1<br>No=0
6.Do you experience problems in your daily life due to poor vision?	Yes=1<br>No=0
7.Do you experience problems in your daily life due to lack of strength in your hands?	Yes=1<br>No=0
8.Do you experience problems in your daily life due to physical tiredness?	Yes=1<br>No=0
9.Do you have problems with your memory?	Yes=1<br>Sometimes=0<br>No=0
10.Have you felt down during the last month?	Yes=1<br>Sometimes=1<br>No=0
11.Have you felt nervous or anxious during the last month?	Yes=1<br>Sometimes=1<br>No=0
12.Are you able to cope with problems well?	Yes=1<br>No=0
13.Do you live alone?	Yes=1<br>No=0
14.Do you sometimes miss having people around you?	Yes=1<br>Sometimes=1<br>No=0
15.Do you receive enough support from other people?	Yes=0<br>No=1

Generalized Self-efficacy（GSES）

	Not at all ture	Barely True	Moderately True	Exactly True
1.I can always manage to solve difficult problems if I try hard enough.	1	2	3	4
2.If someone opposes me. I can find means and ways to get what I want.	1	2	3	4
3.It is easy for me to stick to my aims and accomplish my goals.	1	2	3	4
4.I am confident that I could deal efficiently with unexpected events.	1	2	3	4
5.Thanks to my resourcefulness, I know how to handle unforeseen situations.	1	2	3	4
6.I can solve most problems if I invest the necessary effort.	1	2	3	4
7.I can remain calm when facing difficulties because I can rely on my coping abilities.	1	2	3	4
8.When I am confronted with a problem, I can usually find several solutions.	1	2	3	4
9.If I am in a bind, 1 can usually think of something to do.	1	2	3	4
10.No matter what comes my way, I'm usually able to handle it.	1	2	3	4

Pittsburgh sleep quality index（PSQI）

The following questions relate to your usual sleep habits during the past month only. Your answers should indicate the most accurate reply for the majority of days and nights in the past month. Please answer all questions.

1. During the past month, when have you usually gone to bed at night? Usual bed time:

2. During the past month, how long (in minutes) has it usually take you to fall asleep each night? Number of minutes:

3. During the past month, when have you usually gotten up in the morning? Usual getting up time:

4. During the past month, how many hours of actual sleep did you get at night? (This may be different than the number of hours you spend in bed.) Hours of sleep per night:

For each of the remaining questions, check the one best response. Please answer all questions.

5. During the past month, how often have you had trouble sleeping because you：

Item of the PSQI	Categorical variables
（1）Cannot get to sleep within 30 minutes	Not during the past month
	Less than once a week
	Once or twice a week
	Three or more times a week
（2）Wake up in the middle of the night or early morning	Not during the past month
	Less than once a week
	Once or twice a week
	Three or more times a week
（3）Have to get up to use the bathroom	Not during the past month
	Less than once a week
	Once or twice a week
	Three or more times a week
（4）Cannot breathe comfortably	Not during the past month
	Less than once a week
	Once or twice a week
	Three or more times a week
（5）Cough or snore loudly	Not during the past month
	Less than once a week
	Once or twice a week
	Three or more times a week
（6）Feel too cold	Not during the past month

	Less than once a week
	Once or twice a week
	Three or more times a week
（7）Feel too hot	Not during the past month
	Less than once a week
	Once or twice a week
	Three or more times a week
（8）Had bad dreams	Not during the past month
	Less than once a week
	Once or twice a week
	Three or more times a week
（9）Have pain	Not during the past month
	Less than once a week
	Once or twice a week
	Three or more times a week
	Other reason(s), please describe:

6. During the past month, how would you rate your sleep quality overall?

Very good

Fairly good

Fairly bad

Very bad

7. During the past month, how often have you taken medicine (prescribed or “over the counter”) to help you sleep?

Not during the past month

Less than once a week

Once or twice a week

Three or more times a week

8. During the past month, how often have you had trouble staying awake while driving, eating meals, or engaging in social activity?

Not during the past month

Less than once a week

Once or twice a week

Three or more times a week

9. During the past month, how much of a problem has it been for you to keep up enough enthusiasm to get things done?

No problem at all

Only a very slight problem

Somewhat of a problem

A very big problem

10. Do you have a bed partner or roommate?

No bed partner or roommate

Partner/roommate in other room

Partner in same room, but not same bed

Partner in same bed

11.If you have a roommate or bed partner, ask him/her how often in the past month you have had...

Item of the PSQI	Categorical variables
Loud snoring	Not during the past month
	Less than once a week
	Once or twice a week
	Three or more times a week
Long pauses between breaths while asleep	Not during the past month
	Less than once a week
	Once or twice a week
	Three or more times a week
Legs twitching or jerking while you sleep	Not during the past month
	Less than once a week
	Once or twice a week
	Three or more times a week
Episodes of disorientation or confusion during sleep	Not during the past month
	Less than once a week
	Once or twice a week
	Three or more times a week
Other restlessness while you sleep; please describe	Not during the past month
	Less than once a week
	Once or twice a week
	Three or more times a week

Scoring Instructions for the Pittsburgh Sleep Quality Index

The Pittsburgh Sleep Quality Index (PSQI) contains 19 self-rated questions and 5 questions rated by the bed partner or roommate (if one is available). Only self-rated questions are included in the scoring. The 19 self-rated items are combined to form seven "component" scores, each of which has a range of 0-3 points. In all cases, a score of "0" indicates no difficulty, while a score of "3" indicates severe difficulty. The seven component scores are then added to yield one "global" score, with a range of 0-21 points, "0" indicating no difficulty and "21" indicating severe difficulties in all areas.

Scoring proceeds as follows:

Component 1: Subjective sleep quality

Examine question 6, and assign scores as follows:

Response	Component 1 score
"Very good"	0
"Fairly good"	1
"Fairly bad"	2
"Very bad"	3

Component 1 score:____________

Component 2: Sleep latency

1. Examine question 2, and assign scores as follows:

Response	Score
≤15 minutes	0
16-13 minutes	1
31-60 minutes	2
>60 minutes	3

Question 2 score:______________

2. Examine question 5(a), and assign scores as follows:

Response	Score
Not during the past month	0
Less than once a week	1
Once or twice a week	2
Three or more times a week	3

Question 5(a) score:____________

3. Add 2 score and 5a score

Sum of 2 and 5(a):_____________

4. Assign component 2 score as follows:

Sum of 2 and 5(a)	Component 2 score
0	0
1-2	1
3-4	2
5-6	3

Component 2 score:____________

Component 3: Sleep duration

Examine question 4, and assign scores as follows:

Response	Component 3 score
>7 hours	0
6-7 hours	1
5-6 hours	2
<5 hours	3

Component 3 score:____________

Component 4: Habitual sleep efficiency

(1) Write the number of hours slept (question 4) here:_________
(2) Calculate the number of hours spent in bed:
Getting up time (question 3): ________
Bedtime (question 1): ________
Number of hours spent in bed: ________
(3) Calculate habitual sleep efficiency as follows:
(Number of hours slept/Number of hours spent in bed) *100=Habitual sleep efficiency (%)
(________/________)*100=_________%
(4) Assign component 4 score as follows:

Habitual sleep efficiency %	Component 4 score
>85%	0
75-84%	1
65-74%	2
<65%	3

Component 4 score:____________

Component 5: Sleep disturbances
(1) Examine questions 5(b)-5(j), and assign scores for each question as follows:

Response	Score
Not during the past month	0
Less than once a week	1
Once or twice a week	2
Three or more times a week	3

5 (1)
(b) score:_________
(c) score: _________
(d) score: _________
(e) score: _________
(f) score: _________
(g) score: _________
(h) score: _________
(i) score: _________
(j) score: _________
(2) Add the scores for questions 5(b)-5(j):

Sum of 5(b)-5(j):____________

(3) Assign component 5 score as follows:

Sum of 5(b)-5(j)	Component 5 score
0	0
1-9	1
10-18	2

19-27	3

Component 5 score:____________

Component 6: Use of sleeping medication

Examine question 7 and assign scores as follows:

Response	Component 6 score
Not during the past month	0
Less than once a week	1
Once or twice a week	2
Three or more times a week	3

Component 6 score:____________

Component 7: Daytime dysfunction

(1) Examine question 8, and assign scores as follows:

Response	Score
Never	0
Once or twice	1
Once or twice each week	2
Three or more times each week	3

Question 8 score:______________

(2) Examine question 9, and assign scores as follows:

Response	Score
No problem at all	0
Only a very slight problem	1
Somewhat of a problem	2
A very big problem	3

Question 9 score:______________

(3) Add the scores for question 8 and 9:

Sum of 8 and 9:____________

(4) Assign component 7 score as follows:

Sum of 8 and 9	Component 7 score
0	0
1-2	1
3-4	2
5-6	3

Component 7 score:___________

Global PSQl Score

Add the seven component scores together:

Global PSQI Score:___________
